# Umpolung of a covalent organic framework for high-performance cathodic sodium ion storage†

**DOI:** 10.1039/d5sc01195g

**Published:** 2025-03-25

**Authors:** Fangyuan Kang, Yuchan Zhang, Zihao Chen, Zhaowen Bai, Qianfeng Gu, Jinglun Yang, Qi Liu, Yang Ren, Chun-Sing Lee, Qichun Zhang

**Affiliations:** a Department of Materials Science and Engineering, City University of Hong Kong Tat Chee Avenue 83 Kowloon Hong Kong SAR 999077 P. R. China qiczhang@cityu.edu.hk; b Department of Physics, City University of Hong Kong Tat Chee Avenue 83 Kowloon Hong Kong SAR 999077 P. R. China; c Department of Chemistry, Center of Super-Diamond and Advanced Films (COSDAF), Hong Kong Institute of Clean Energy (HKICE), City University of Hong Kong Hong Kong SAR 999077 P. R. China apcslee@cityu.edu.hk; d City University of Hong Kong Shenzhen Research Institute Shenzhen Guangdong Province 518057 P. R. China

## Abstract

The rational design of electrode materials to modify their intrinsic electronic states effectively enhances the performance of rechargeable batteries. Herein, an umpolung strategy is implemented in preparing a polyimide-linked COF (CityU-47) through a polar inversion of the typical p-type triphenylamine (TPA) with a multi-carbonyl-contained n-type azatriangulenetrione (ATTO). This strategy can substantially decrease the energy level of the lowest unoccupied molecular orbital (LUMO), thereby increasing the potential for operation as a cathode material. Alongside increased specific capacity, an improved overall performance in sodium-ion batteries (SIBs) is achieved. Specifically, CityU-47 provides a high capacity of 286.31 mA h g^−1^ at a current density of 0.1 A g^−1^, and a cycle capacity of 210 mA h g^−1^ at 2 A g^−1^ over 1800 cycles is also achieved. This research offers fresh perspectives on enhancing battery performance, underscoring the importance of regulating electron structures at the atomic level.

## Abstract 2

Umpolung of the typical p-type TPA into n-type ATTO contributes to the COF electrode with a remarkable cathodic capacity of 286.31 mA h g^−1^ at a current density of 0.1 A g^−1^ in SIBs.

## Graphical abstract

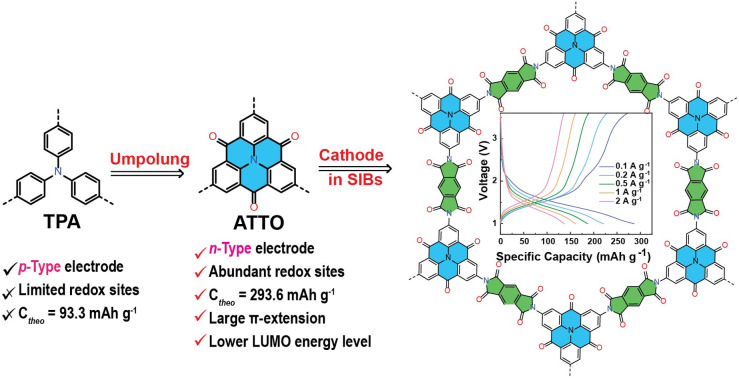

## Introduction

In the “beyond-lithium-storage” stage of development, sodium-ion batteries (SIBs), with their abundant resources and similar intercalation mechanism to lithium-ion batteries (LIBs), have emerged as key players, particularly for large-scale energy storage facilities and low-speed electric vehicles.^1–3^ However, several issues mean SIBs are far from practical application.^4,5^ One major issue is the lower specific capacity of SIBs compared to LIBs due to the much larger atomic weight of Na^+^ compared to Li^+^. Additionally, Na^+^/Na shows a ∼300 mV higher redox potential than Li^+^/Li (−2.71 V *vs.* −3.05 V). As a result, SIBs inherently have a lower energy density by at least 30% compared to LIBs. The energy density is a result of the specific capacity and average working voltage. The operating voltage of electrodes is primarily determined by the local electronic structures and the state of electrons participating in redox reactions,^6,7^ which can be manipulated using the molecular structure at the atomic level. For example, by introducing electron-withdrawing groups such as –F, –Cl, and –CN into electrode materials, the redox potential can be improved due to the decrease of the lowest unoccupied molecular orbital (LUMO) energy level.^8–12^ However, this may lead to a cost in terms of the theoretical specific capacity. Furthermore, the larger radius of Na^+^ (1.02 Å *vs.* 0.76 Å of Li^+^) results in sluggish kinetics, considerable volume variation, poor rate performance, and inferior cyclability in SIBs. Conventional inorganic electrode materials struggled to address these challenges until the recently emerged highly designable covalent organic framework (COF) materials,^13,14^ providing the possibility to meet the requirements in SIBs.^15–18^

COFs are a class of ordered porous polymers assembled from pre-designed building blocks through strong covalent bonds,^19,20^ which were first utilized as electrodes in SIBs by Xu and co-workers in 2018.^21,22^ When compared to inorganic and traditional organic electrode materials, COF electrodes offer numerous distinct advantages and unique characteristics that make them ideal candidates for SIBs.^23^ They feature well-defined one-dimensional (1D) nanoporous channels, and Na^+^ guests with larger radii can easily and swiftly percolate during the redox processes. They also feature adjustable pore sizes, and thermal and chemical robustness of the frameworks, preventing uncontrollable volume change and structural collapse of the COFs.^24^ Meanwhile, the high structural designability enables the direct integration of a wide range of tailorable redox-active functionalities to increase the theoretical specific capacity and the average output voltage.^25,26^

Organic electrode materials can be roughly divided into n-, p-, and bipolar types.^27–29^ Typically, p-type organic electrodes deliver higher redox potentials but limited capacity due to the low density of redox-active groups. Conversely, n-type organic electrodes generally provide high capacity but have a lower working voltage.^24,30^ Therefore, overcoming this capacity–potential trade-off is crucial for achieving high-energy-density COF electrodes. As the redox potential is intrinsically related to the functional groups and molecular skeletons,^16,31^ COFs benefit from high customization of the skeleton and functionalities, which allows for a high potential to simultaneously increase their theoretical specific capacity and working potential by meticulous structural design. However, this avenue has not been well explored in the field of COFs.^7^

**Fig. 1:**
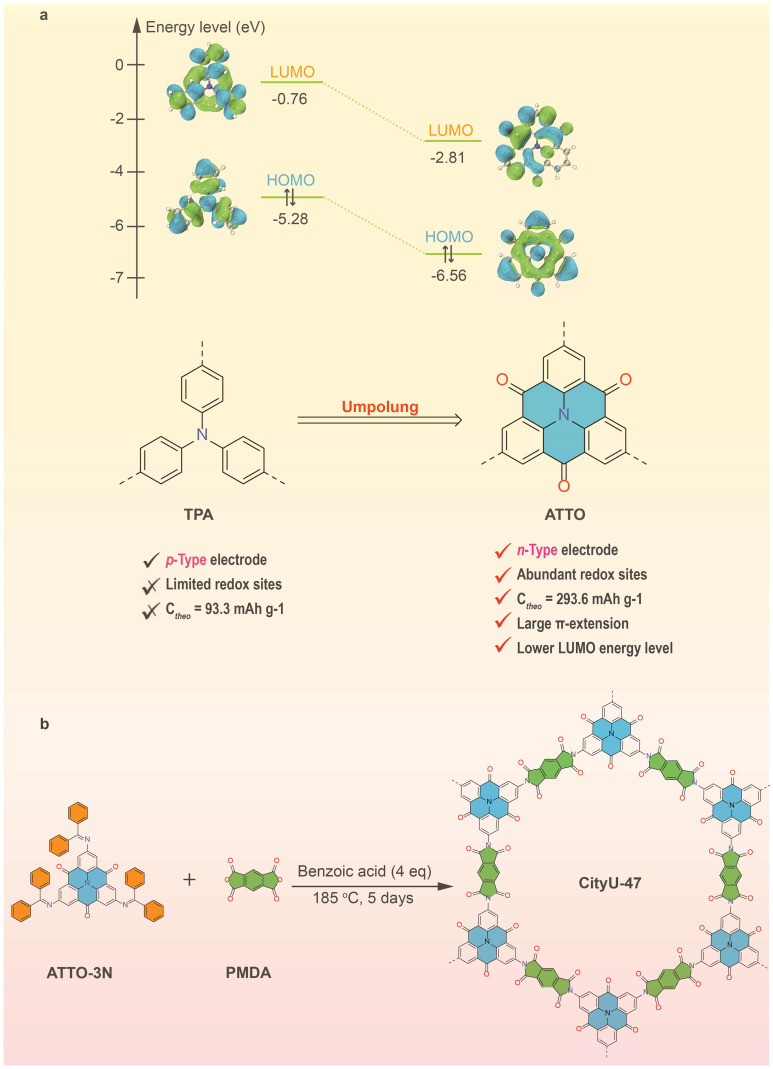
Illustration of umpolung. (a) Design principle of structural umpolung with TPA, and (b) integration of the umpolung unit into the COF.

Recently, carbonyl groups have been frequently enrolled as electroactive centers in COFs to contribute to the specific capacity of batteries.^32,33^ The strong electron-withdrawing effect of the carbonyl moiety also influences the electronic distribution in the frameworks, leading to a lower LUMO energy level and an enhanced redox potential (Fig. 1a).^34,35^ In a novel approach, a common p-type triphenylamine (TPA) has been transformed into a carbonyl-enriched n-type azatriangulenetrione (ATTO) unit through umpolung to synthesize a polyimide-linked COF known as CityU-47 (Fig. 1a and b). This strategic polar inversion at the atomic level offers several benefits: (1) the dense redox-active groups of the carbonyl units in ATTO and polyimide linkages directly act as compensators of specific capacity to contribute to the remarkable capacity of CityU-47; (2) the robust polyimide linkages also ensure structural stability during the cycling; (3) the electron-withdrawing effect of the carbonyl groups significantly decreases the LUMO energy level and reduces the band gap, resulting in the increased working potential and the enhanced electrical conductivity;^36–38^ and (4) by comparing the single-crystal structures of TPA and ATTO derivatives,^25,39^ it is evident that ATTO exhibits higher planarity, larger π-extension, and better overlap of π electron clouds. These characteristics enhance the redox potential and electron transport ability of the resulting COF electrode, thereby improving the rate performance.^40–42^ Indeed, CityU-47 has been demonstrated to possess a superior capacity of 286.31 mA h g^−1^ at a current density of 0.1 A g^−1^, with an increased cycle capacity of over 200 mA h g^−1^ achieved after 1800 cycles at 2 A g^−1^. This research showcases a promising COF design strategy for enhancing the performance of SIBs.

## Results and discussion

Polyimide-linked COFs are typically synthesized using solvothermal methods at high temperatures, primarily due to the low reversibility of the imidization reaction. However, this approach is environmentally unfriendly due to the use of toxic organic solvents and catalysts such as isoquinoline.^43,44^ In contrast, the recently developed organic flux synthesis has emerged as an effective alternative with a broad range of substrates.^45–47^ Due to the enhanced solubility of ATTO-3N in flux through a protective strategy, crystalline CityU-47 can be directly synthesized by reacting ATTO-3N with pyromellitic dianhydride (PMDA) at 185 °C for 5 days within the flux of equivalent benzoic acid (Fig. 1b). The resulting monolith was then subjected to a Soxhlet extraction with dimethylformamide/tetrahydrofuran for 3 days, followed by an exchange with acetone and *n*-hexane. Subsequently, the product was dried at 120 °C under vacuum for 12 h for further characterization and application.

**Fig. 2:**
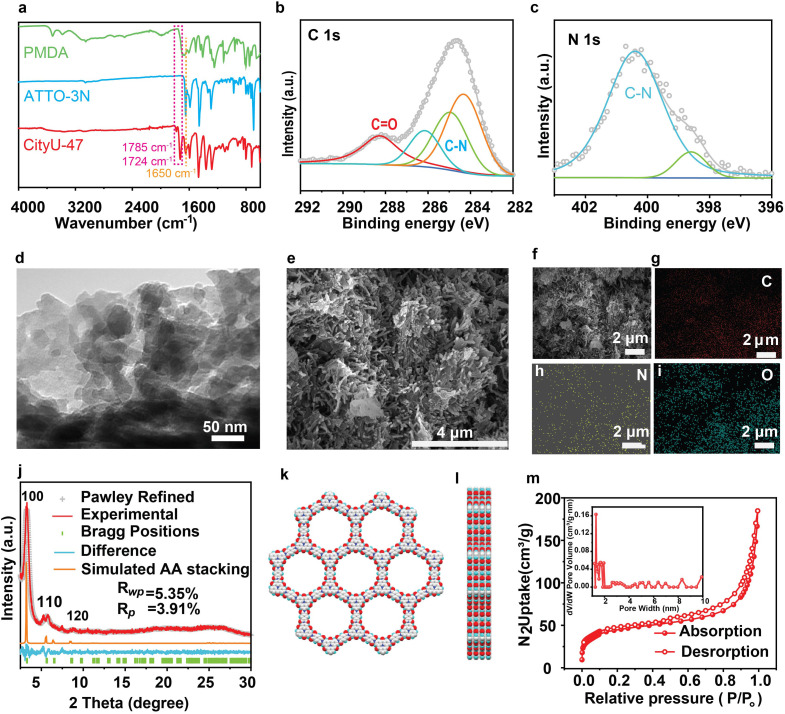
Structural characterizations of CityU-47. (a) FTIR spectra of CityU-47 and starting materials. XPS profiles of CityU-47: (b) C 1s and (c) N 1s. (d) TEM images, and (e) SEM images. (f–i) EDX mapping images of C, N, and O. (j) PXRD patterns of experimental (red), Pawley-refined (grey), and simulated stacking (orange) results, and their difference (light blue), and Bragg positions (green). (k and l) Space-filling model of AA stacking. (m) Nitrogen adsorption–desorption isothermsat 77 K.

Fourier transform infrared (FTIR) and X-ray photoelectron spectroscopy (XPS) techniques were employed to confirm the structure and composition of the as-synthesized CityU-47. Following the flux process, the FTIR spectra display distinct asymmetric and symmetric vibrations of the C=O groups in the imide structure at 1785 cm^−1^ and 1724 cm^−1^, respectively. Additionally, the original signal of the C=O group in ATTO-3N at ∼1650 cm^−1^ is well preserved, indicating the successful formation of the targeted polyimide-linked CityU-47 (Fig. 2a). In the XPS analysis (Fig. S1a†), the C 1s spectrum exhibits peaks at binding energies of 284.5, 285, 286.5, and 288.7 eV, corresponding to the groups of C=C, C–C, C–N, and C=O (Fig. 2b), respectively. Moreover, peaks assigned to C–N in the N 1s spectrum (Fig. 2c) and C=O in the O 1s spectrum (Fig. S1b†) are observed at 401 eV and 531.6 eV, respectively, providing further evidence to support the successful construction of CityU-47. Transmission electron microscopy (TEM) and scanning electron microscopy (SEM) techniques were utilized to investigate the morphology of CityU-47, revealing dense rodlike aggregates that are consistent with its monolithic properties (Fig. 2d and e). Moreover, EDX mapping results show a good distribution of C, N, and O elements (Fig. 2f–i).

Powder X-ray diffraction (PXRD) analysis was conducted to assess the crystallinity (Fig. 2j). The PXRD pattern exhibits a sharp diffraction peak at 3.2°, along with smaller peaks at 5.7° and 8.7° corresponding to the 100, 110, and 120 facets, respectively, indicating the high crystallinity of CityU-47. Structural simulation and Pawley refinement by BIOVIA Materials Studio implied that CityU-47 adopted an AA stacking mode in the space group *P*1 (*R*_wp_ = 4.52% and *R*_p_ = 3.52%; cell parameters: *a* = *b* = 31.77 Å, *c* = 3.67 Å, and *α* = *β* = 90°, *γ* = 120°) (Fig. 2j–l). Nitrogen adsorption/desorption measurements were performed at 77 K to measure the porosity features. As shown in Fig. 2m, CityU-47 displays a typical type-I reversible isotherm with a calculated Brunauer–Emmett–Teller (BET) surface area of 154.8 m^2^ g^−1^. Thermogravimetric analysis (TGA) demonstrated that CityU-47 possesses good thermal stability, with a decomposition temperature exceeding 458 °C with a weight loss of 5% under a nitrogen atmosphere (Fig. S2†).

**Fig. 3:**
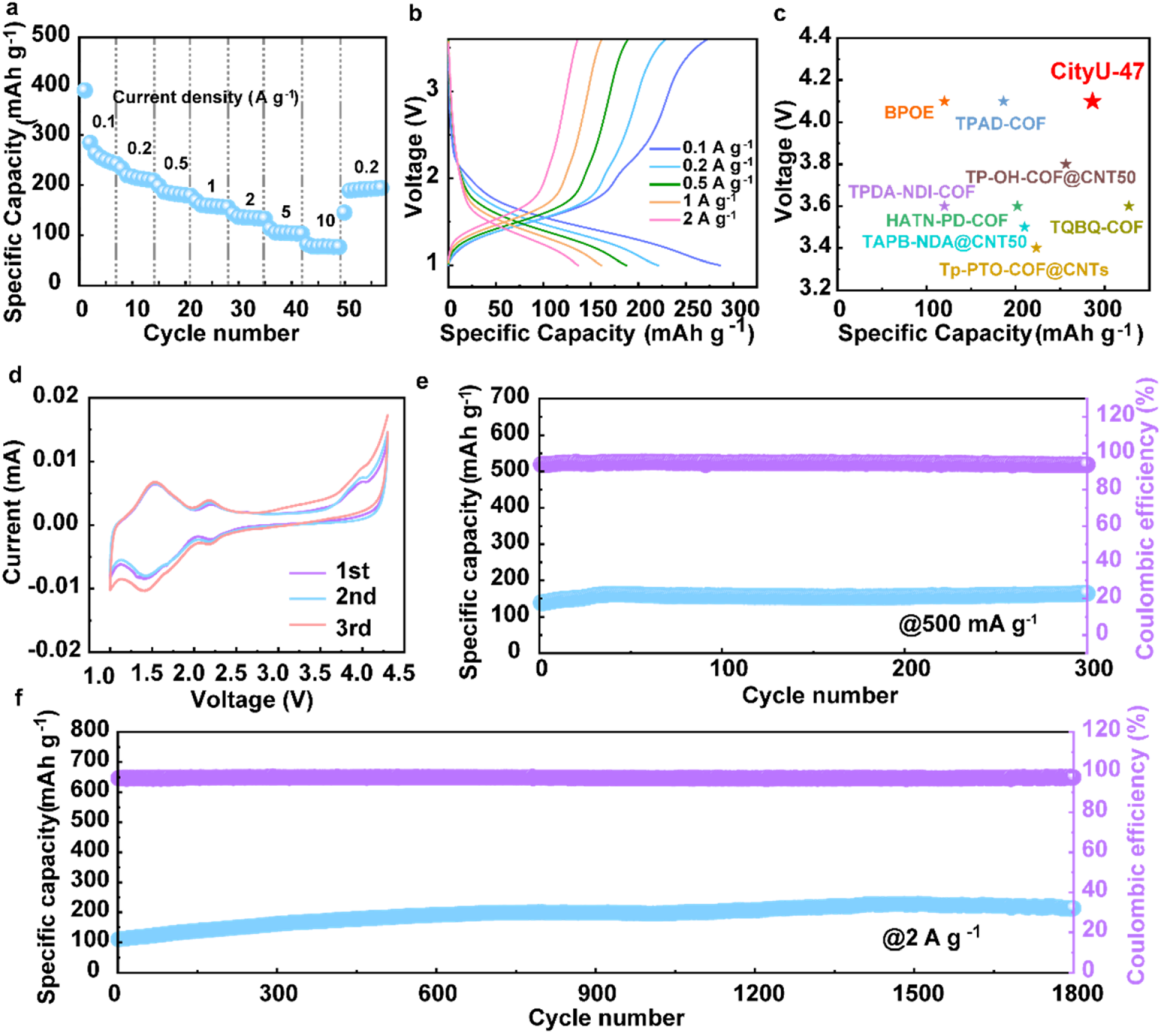
Electrochemical evaluation of CityU-47. (a) Rate capability performance. (b) Charge–discharge voltage profiles at different current densities. (c) Performance comparison of different organic cathodes in SIBs. (d) CV curves at 0.1 mV S^−1^. (e) Cyclic performance at 500 mA g^−1^. (f) Long cyclic performance at 2 A g^−1^.

To elucidate the umpolung method in improving device performance, CityU-47 was used as the cathode for a Na ion battery. CR2032 coin cells were assembled to probe the de-intercalation ability of Na ions in rechargeable batteries. CityU-47 delivers rate capacities of 286.31, 234.73, 199.43, 169.12, 143.56, 112.79 and 81.12 mA h g^−1^ at current densities of 0.1, 0.2, 0.5, 1, 2, 5 and 10 A g^−1^, respectively (Fig. 3a). Meanwhile, when the current density returns to 200 mA g^−1^, the capacity can be restored to its initial level, implying the excellent reversibility in the storage of Na ions.^15^ The charging–discharging curves at different current densities are shown in Fig. 3b, which displays typical discharge platforms at 2–2.2 V and 1.3–1.5 V. Compared to previously reported organic cathodes,^4,48–54^ CityU-47 is placed among the top materials, showcasing the effectiveness of the umpolung strategy in improving battery performance (Fig. 3c).

Cyclic voltammetry (CV) analysis was also conducted (Fig. 3d), and provided results consistent with those in Fig. 3b. The overlapping profiles of the CV curves in the first three cycles at a scan rate of 0.1 mV s^−1^ suggest that CityU-47 can stably and reversibly accommodate Na ions.^26^ In the scan curves, the cathodic peaks at around 2.2 V and a broad peak at 1.4 V are attributed to the insertion of Na ions into CityU-47. Moreover, the anodic peaks at around 1.5 and 2.2 V point towards CityU-47 having a two-step insertion and extraction process for Na ions. The cycling performances were also recorded. When cycling at 200 mA g^−1^ (Fig. S3†), CityU-47 displays a high specific capacity of ∼260 mA h g^−1^ after 100 cycles. For operations at high current densities, a pre-cyclic process for activation was implemented.^55^ After this process, the specific capacity remained stable at around 170 mA h g^−1^ for 500 cycles at a current density of 500 mA g^−1^ (Fig. 3e). Impressively, when the current density was 2 A g^−1^, CityU-47 achieved an increased capacity of over 210 mA h g^−1^ after 1800 cycles (Fig. 3f), demonstrating the good stability of COF materials in SIBs. The high-temperature cycling performance was also tested (Fig. S4–S6†). CityU-47 displayed a consistent ion storage capacity at 60 °C and maintained a cycling capacity of over 100 mA h g^−1^ at a current density of 2 A g^−1^ after 200 cycles.

**Fig. 4:**
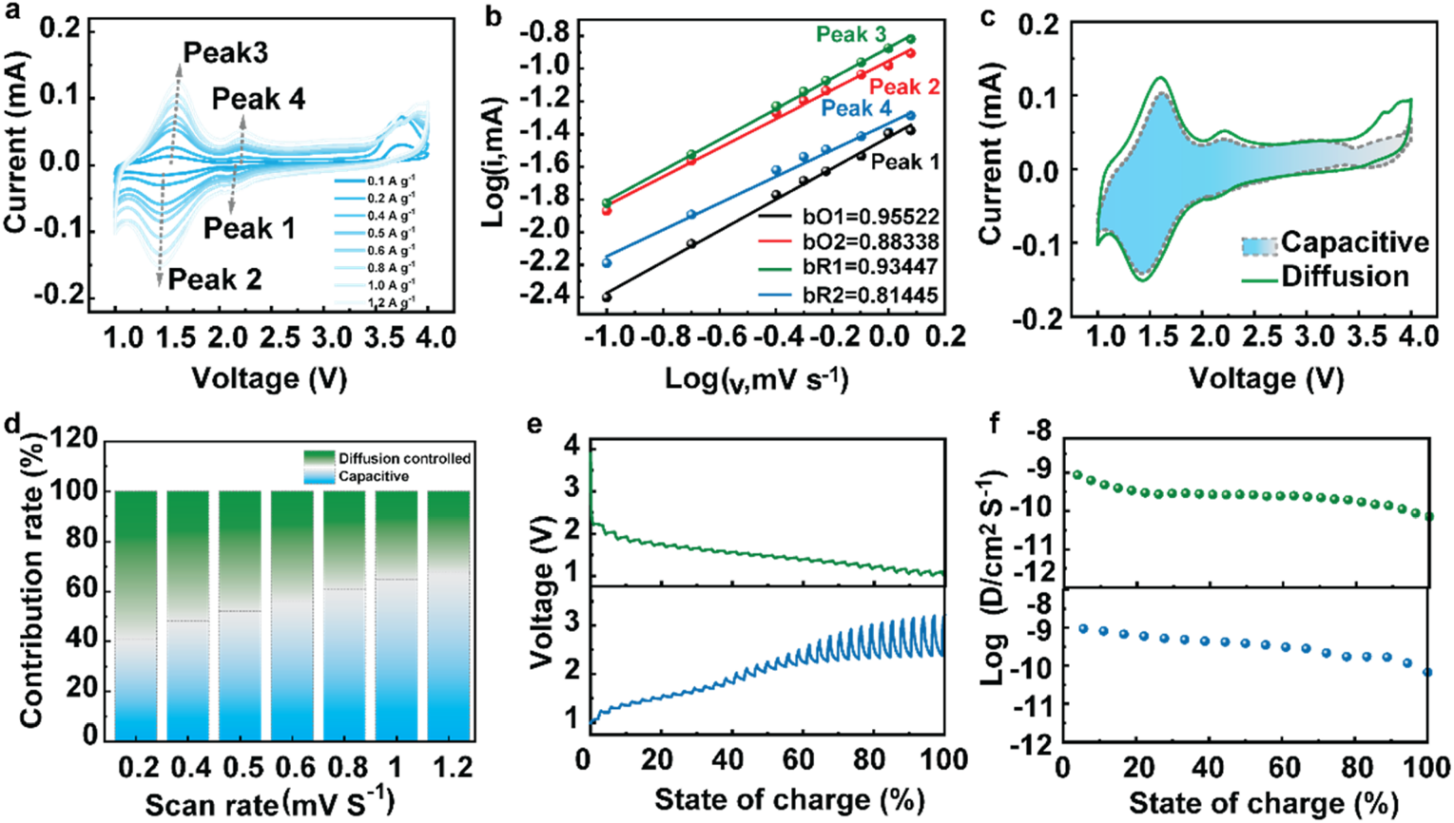
Kinetic tests of CityU-47. (a) CV curves at various scan rates. (b) Plots of the b value at different peak rates. (c) The capacitive contribution of the CV curves. (d) The capacitive contribution with the proportions at different scan rates. (e) GITT curves of charging and discharging. (f) The corresponding *D*_Na^+^_ for GITT.

To understand the mechanistic behavior of the COF cathode, a variety of kinetic tests were conducted. Fig. 4a displays the CV profiles at various scan rates of 0.1, 0.2, 0.4, 0.5, 0.6, 0.8, 1.0 and 1.2 mV s^−1^. As the scan rate was incrementally increased, the curves retained their consistency and the peaks experienced minor shifts. This suggests that the charge transfer is rapid and the electrochemical kinetics are significantly enhanced. Fig. 4b displays the profiles of the currents of peaks and scan rates. The *b* value is calculated using the formula:

1 *i* = *av*^*b*^

2 log *i* = *b* log *v* + log *a*

If the *b* value approaches 1, it means that the electrochemical performance is a surface-controlled process. If *b* approaches 0.5, it means that the reaction is a diffusion-controlled process. The *b* values of the four peaks are 0.96, 0.88, 0.92 and 0.81, suggesting that both processes contribute to the electrochemical reaction of CityU-47. The capacitive contribution of the CV curves is exhibited in Fig. 4c. According to the calculation at different scan rates using the formula *I* = *k*_1_*v* + *k*_2_*v*^1/2^, the pseudocapacitive contribution at 1.2 mV s^−1^ was about 69% of the total capacity. With the increase of the scan rate from 0.2 to 1.2 mV s^−1^, the contribution of surface-controlled capacitance increased from 42% to 69%, suggesting that CityU-47 possesses good reversibility.^16^ In addition, in order to estimate the solid-state diffusion coefficient of Na ions, the galvanostatic intermittent titration technique (GITT) and Na ion diffusion coefficient measurement were conducted, and the results are shown in Fig. 4e and f. The Na ion diffusion coefficients (*D*_Na^+^_) were calculated at different charging/discharging states according to the following formula:*D* = 4*L*^2^/π*τ*(Δ*E*_S_/Δ*E*_t_)^2^

Using the calculation, the *D*_Na^+^_ value of the CityU-47 cathode was determined to be 10^−9^ cm^2^ S^−1^. Clearly, the high diffusion coefficient implies that CityU-47 has high Na ion mobilities, and also significantly contributes to its superb electrochemical capability.^26^*Ex situ* EIS at different charge and discharge voltages was performed (Fig. S7†). The impedance values show a moderate increase and decrease during Na insertion/extraction, indicating the stable surface of this COF cathode.

**Fig. 5:**
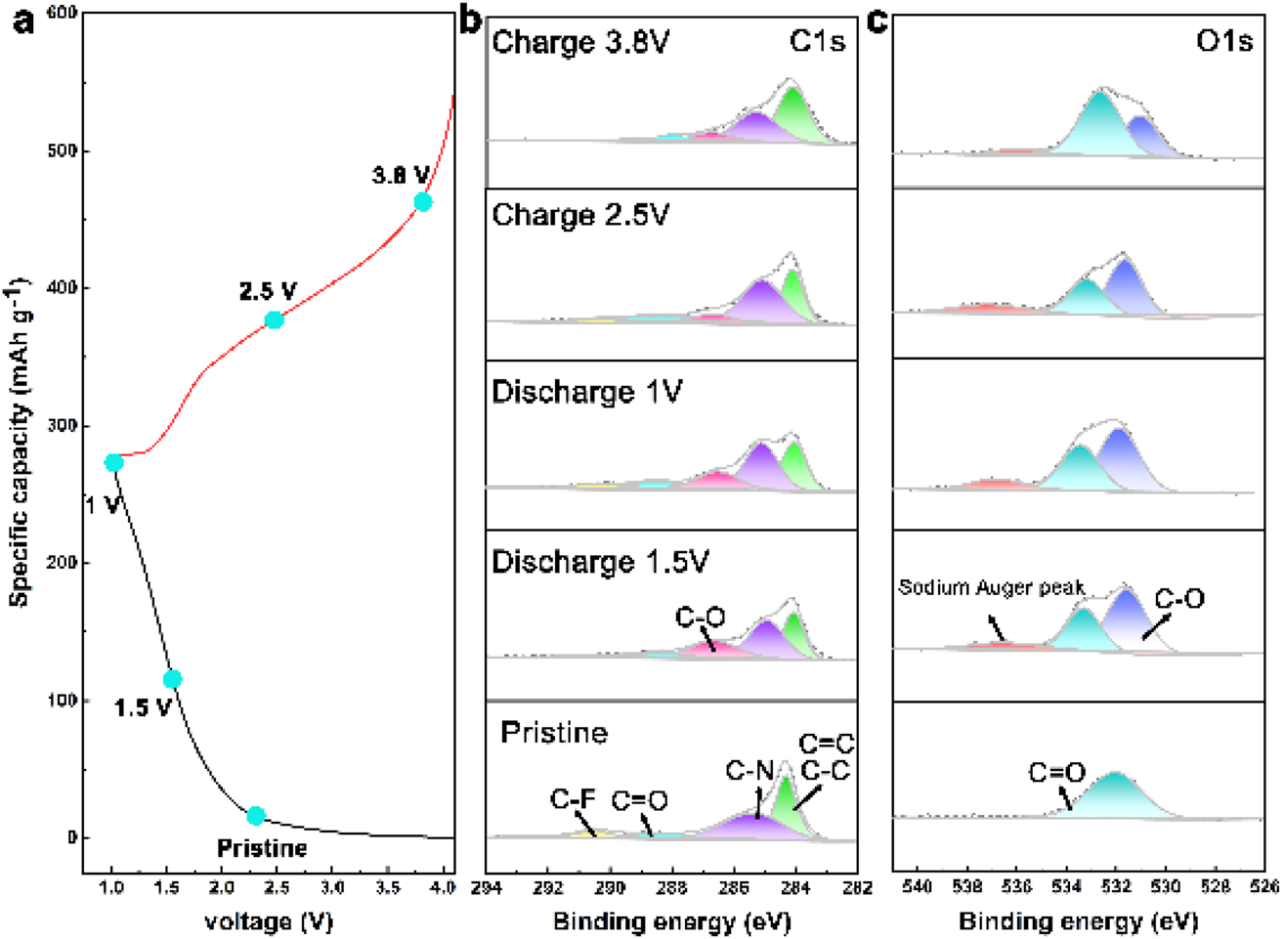
Mechanistic behavior of CityU-47. (a) Charge–discharge curves. (b and c) *Ex situ* XPS spectra of C 1s and O 1s.

To delve deeper into the mechanisms of Na ion intercalation/deintercalation during cycling, *ex situ* XPS, FTIR, and density functional theory (DFT) calculations were carried out at different states. The *ex situ* XPS spectra (Fig. 5b and c) show that the C=O peak gradually decreases when discharged to 1.5 V and 1.0 V. Simultaneously, the C–O peak gradually emerges and intensifies during the discharge process, and diminishes after charging to 2.5 V and 3.8 V, suggesting a reversible conversion between C=O and C–O⋯Na. Significant changes were also observed in the O 1s spectrum (Fig. 5c). The C=O peaks decreased and C–O peaks were enhanced during the discharge process. Moreover, the appearance of sodium Auger peaks during sodiation/desodiation confirms the ability of the COF cathode to store Na ions.

The *ex situ* FTIR spectra at different discharge and charge states are depicted in Fig. S8 and S9.† Compared to the pristine spectra, the characteristic peaks of C–C without active groups remained relatively unchanged during the charging and discharging states, indicating the skeletal integrity of the COF during electrochemical reactions. Notably, the intensity of the stretching vibration of the C=O group gradually weakens upon discharging to 1.5 V and 1.0 V. It is renewed upon charging to 2.5 and 3.8 V. This suggests the reversible Na^+^ storage ability of C=O groups in CityU-47 and is consistent with the XPS spectra.

**Fig. 6:**
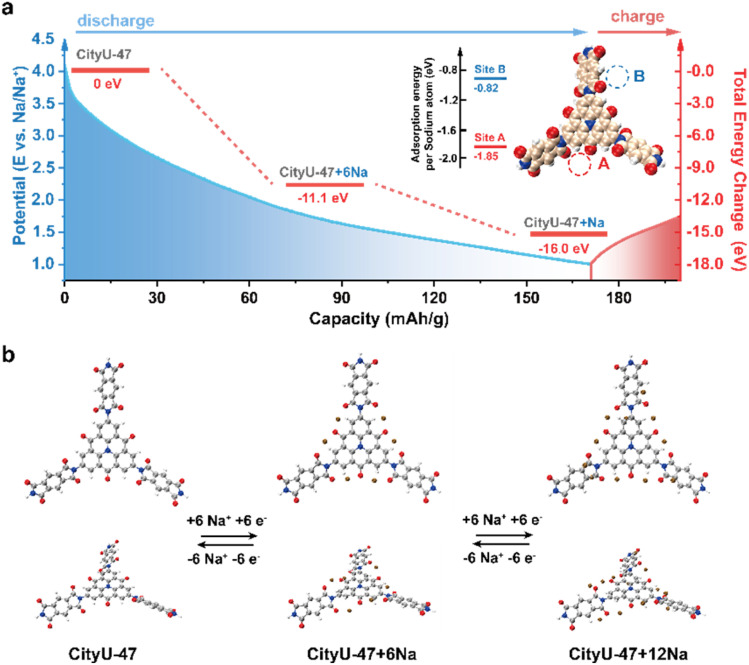
DFT calculations. (a) Proposed sodiation pathway of the CityU-47 electrode, with redox potential *vs.* Na/Na^+^ on the left axis and total energy change on the right axis. (b) The corresponding structural evolution of CityU-47 during the sodiation procedure.

Density functional theory (DFT) calculations were conducted to further elucidate the structural evolution of CityU-47. Using the repeating unit of CityU-47 as the theoretical model, the calculated lowest unoccupied molecular orbital (LUMO) and highest occupied molecular orbital (HOMO) indicated an energy gap of 3.05 eV.^56^ The distribution of the HOMO and LUMO revealed that ATTO functions as a donor while the polyimide unit acts as an acceptor in the framework (Fig. S10†). This donor–acceptor (D–A) system enhances the electrical conductivity, facilitates charge transport, and accelerates redox kinetics, contributing to the superior rate performance of CityU-47.^57,58^ The molecular electrostatic potential (MESP) distribution illustrated the formation of continuous electron-rich regions due to the dense distribution of carbonyl functional groups in the backbone of CityU-47 (Fig. S11†), enabling the rapid approach of Na ions to the redox-active sites. To understand the position and order of sodium ion insertion, sodiation models were constructed, and the adsorption energies of Na atoms at each site were calculated. As shown in Fig. 6a, two adjacent carbonyl groups interact cooperatively with a single Na ion at Site A, yielding an adsorption energy of −1.85 eV. In contrast, at Site B, a single carbonyl group interacts with a Na ion, resulting in an adsorption energy of −0.82 eV. These two types of adsorption site correspond to the two redox peaks observed in Fig. 4a. Further analysis of the total energy variation during the Na-ion insertion process was carried out as shown in Fig. 6b. Initially, six Na ions bind to the six A sites in the CityU-47 structure, forming the CityU-47-6Na configuration with a binding energy of −11.1 eV. Subsequently, an additional 6 Na ions are intercalated into the B sites, resulting in the CityU-47-12Na structure with a binding energy of −16.0 eV. These steps aligned with the voltage ranges of 4.1–2.2 V and 2.2–1.0 V during the discharge process.

## Conclusions

The components in COFs, such as building blocks, linkages, functional groups, conjugation units, *etc.*, can be strategically tailored to optimize their electrochemical parameters. This study incorporated a carbonyl-enriched electroactive unit (ATTO) into a polyimide-linked COF (CityU-47) following an umpolung from a TPA moiety. This structural inversion provides CityU-47 with enhanced capacity, conjugation, and planarity when utilized as a cathode in SIBs. Consequently, 12 Na^+^ can be inserted into one repeating unit of CityU-47, resulting in a high capacity of up to 286.31 mA h g^−1^ at a current density of 0.1 A g^−1^. CityU-47 also exhibited excellent cycle stability, maintaining steady cycling for 1800 cycles at 2 A g^−1^. The umpolung strategy employed in this study offers a promising perspective for COF designers aiming to improve device performance.

## Author contributions

C. S. Lee and Q. Zhang designed the research. F. Kang and Y. Zhang conducted the experiments. Z. Chen finished the DFT calculations. Z. Bai, Q. Feng, and J. Yang conducted the structural characterizations. Q. Liu and Y. Ren provided suggestions for the electrochemical tests.

## Conflicts of interest

There are no conflicts to declare.

## Supplementary Material

SC-016-D5SC01195G-s001

## Data Availability

All experimental procedures and characterisation data can be found in the article or in the ESI.†
