# Supplementary material for: Umpolung of a covalent organic framework for high-performance cathodic sodium ion storage
Source: Chem Sci. 2025 Mar 25;16(18):7711–9. doi: 10.1039/d5sc01195g (PMC11960781; doi:10.1039/d5sc01195g)
Supplement: SC-016-D5SC01195G-s001 [file SC-016-D5SC01195G-s001.pdf]

Supplementary Information (SI) for Chemical Science.
This journal is © The Royal Society of Chemistry 2025

Umpolung of Covalent Organic Framework Towards High-Performance Cathodic Sodium Ions Storage

Fangyuan Kang,[a]† Yuchan Zhang,[a]† Zihao Chen,[a] ,Zhaowen Bai,[b] Qianfeng Gu,[a] Jinglun Yang,[a] Qi Liu,[b] Yang Ren,[b] Chun-Sing Lee,*[c] Qichun Zhang*[a][c][d]

[a] Dr. F. Kang, Dr. Y. Zhang, Z. Chen, Q. Gu, J. Yang, Prof. Q. Zhang
Department of Materials Science and Engineering
City University of Hong Kong
Tat Chee Avenue 83, Kowloon, Hong Kong SAR, 999077, P. R. China
E-mail: qiczhang@cityu.edu.hk

[b] Dr. Z, Bai, Prof. Q, Liu, Prof. Y, Ren
Department of Physics
City University of Hong Kong
Tat Chee Avenue 83, Kowloon, Hong Kong SAR, 999077, P. R. China

[c] Prof. C-S Lee, Prof. Q. Zhang
Department of Chemistry, Center of Super-Diamond and Advanced Films (COSDAF) &Hong Kong Institute of Clean Energy (HKICE)
City University of Hong Kong
Hong Kong SAR, 999077, P. R. China
E-mail: apcslee@cityu.edu.hk

[d] Prof. Q. Zhang
City University of Hong Kong Shenzhen Research Institute
Shenzhen, Guangdong Province, 518057, P. R. China

† Equal contribution authors

Experimental Parts

ATTO-3N was prepared according to the reported procedures.[1]

All chemicals were used as received without further purification. Pyromellitic dianhydride (PMDA, 99.8%) was purchased from Sigma-Aldrich. Benzoic acid was bought from Shanghai Macklin Biochemical Co. Ltd. Methyl 2-iodobenzoate and methyl 2-aminobenzoate were purchased from J&K company. Diphenylmethanimine and Tetrakis(triphenylphosphine)palladium(0) were bought from Energy Chemical. All other solvents and common inorganic bases were purchased from Anaqua (Hong Kong) Company Limited and used as received without further purification.

Instruments

The Fourier transform infrared (FT-IR) spectra were recorded in the range of 4,000-400 cm^{-1} on a PerkinElmer Spectrum two FT-IR system with $LiTaO_3$ as the detector.

Nitrogen physisorption analyses were conducted on Micromeritics ASAP 3020 equipment and the measurements were performed at 77 K, maintained by a liquid nitrogen bath with pressures ranging from 0 to 760 Torr.

Thermal gravimetric analyses (TGA) were performed on a PerkinElmer Simultaneous Thermal Analyzer ATA 6000 under nitrogen flow (20 mL/min) from ambient temperature to 800 °C at the rate of 10 °C min^{-1}, where 3-6 mg of samples were used and put in a ceramic crucible during the TGA test.

Powder X-ray diffraction patterns were recorded on a Rigaku X-ray Diffractometer SmartLabTM 9kW with Cu–$K_{\alpha 1}$ radiation (λ = 1.5406 Å) by depositing the powder on quartz glass substrate, from 2θ = 3° up to 30° with 0.01° increment.

X-ray photoelectron spectroscopy (XPS) experiments were carried out on an AXIS Ultra DLD system from Kratos with mono Al Kα radiation (1486.6 eV) as X-ray source. Samples were measured under ultra-high vacuum. The existent elements on steel surface were obtained from wide-scan XPS spectra, and the chemical states of these elements were obtained from the high-resolution spectra. In this study, the binding energy of C 1s at 284.6 eV was used to calibrate other binding energies. After

calibrating the binding energies for each element, the fitting of XPS data was carried on with software XPSPEAK. Background line was carefully chosen under Shirley background mode, then different peak positions were fixed and optimized full width at half maxima) and area to fit with a suitable $\sum x2$ value.

Scanning electron microscopy (SEM) measurements were performed on a FEI Sirion-200 field emission scanning electron microscope.

Transmission electron microscopy (TEM) imaging was performed Philips Technai 12, operated at 80 kV.

Structure modeling: The structure of the CityU-47 was constructed using Materials Studio (MS), and the geometry and unit cell were optimized by Forcite method. The general force field and quasi Newton algorithm were used for calculation. The PXRD simulation was carried out using MS modeling.

The synthesis of CityU-47

Pyromellitic Dianhydride (PMDA) (0.09 mmol, 20 mg), ATTO-3N (0.061 mmol, 52.6 mg), and benzoic acid (0.37 mmol, 44.79 mg) were added into a Pyrex tube. The tube was degassed and sealed under vacuum, and heated at 185 °C for 5 days. The resultant monolith was treated with Soxhlet extraction by dimethylformamide/tetrahydrofuran for 3 days, then exchanged with n- hexane. After that, the product was dried at 120 °C under vacuum for 12 h for further characterization and application (yield: 32 mg).

Electrode preparation

The COF-based electrode was prepared through thoroughly mixing CityU-47, Super P and PVDF (3wt% NMP solution) in a weight ratio of 6:3:1 with continuous stirring 12 h until it formed a homogeneous slurry. The slurry was cast into a uniform film on Al current collector and dried at 80 °C for 12 h in a vacuum oven. The mass loading of CityU-47 on the electrode is about 0.6 mg cm^{-2}.

Electrochemical tests

The electrochemical performance of the CityU-47 electrodes including galvanostatic charge/discharge, CV and EIS were evaluated in CR2032-type coin cells with sodium disks as the counter electrodes, where 1 M $NaPF_6$ dissolved in diglyme was used as the

electrolyte, and glass-fiber filter was used as separator. Galvanostatic charge/discharge measurements, rate performance and GITT tests were performed on a Neware CT-4008 battery test system at room temperature and 60°C. The CV measurements at different scan rates and EIS test at different voltage were conducted on the Lvium Software electrochemical workstation.

Density Functional Theory Calculations

All calculation results were calculated using GaussView6.0 and Gaussian16. The structure of CityU-47 was fully optimized and analyzed for the vibrational frequency at B3LYP/6-311G(d) level by Gaussian16. All calculations were performed using DFT-D3(BJ) dispersion correction to describe the dispersion of structure. The visualization image was performed by Multiwfn and VMD software.

Supporting Figures

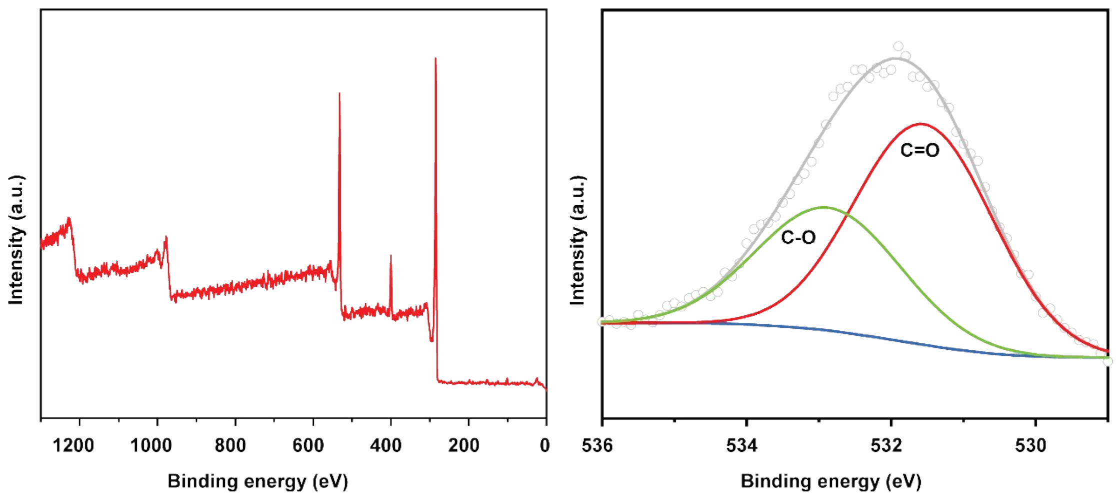

Fig.S1 Full XPS pattern of CityU-47 (a); O 1s XPS pattern (b).

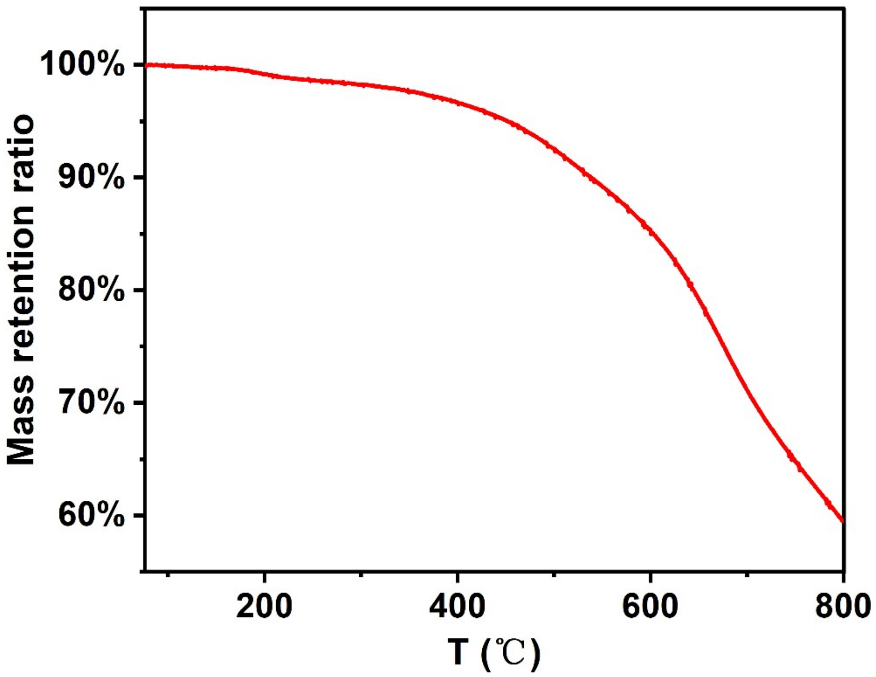

Fig.S2 TGA result of CityU-47.

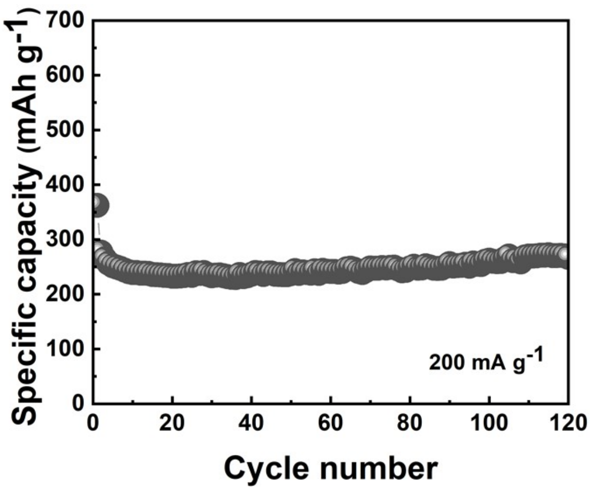

Fig.S3 The cycling performance of CityU-47 at 200 mA g^{-1}.

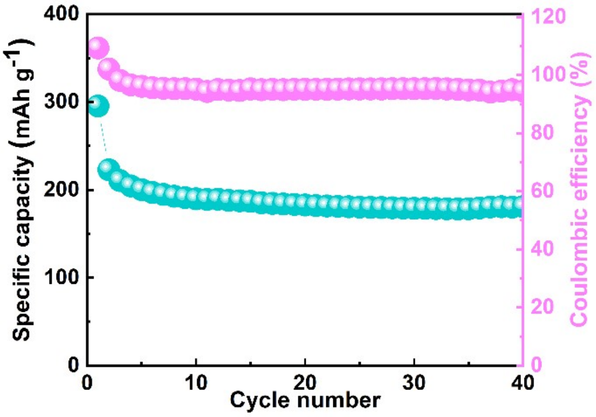

Fig.S4 The cycling performance of CityU-47 at 60 °C at the current density of 200 mA g^{-1}.

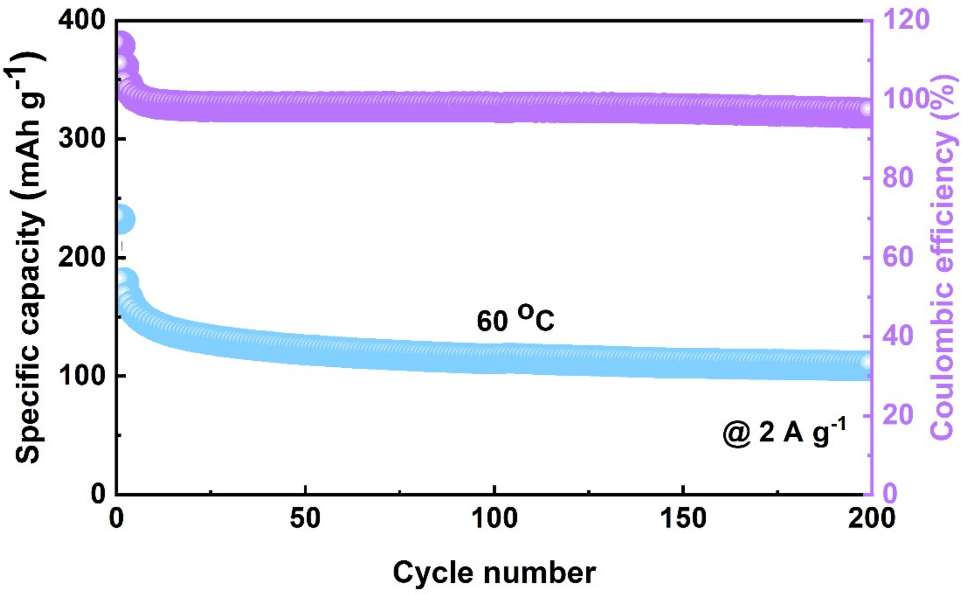

Fig.S5 The cycling performance of CityU-47 at 60 °C at the current density of 2 A g^{-1}.

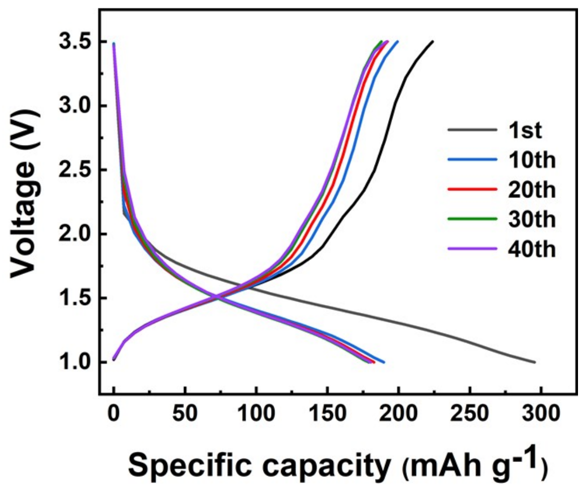

Fig.S6 The charge/discharge curves of CityU-47 at 60 ℃.

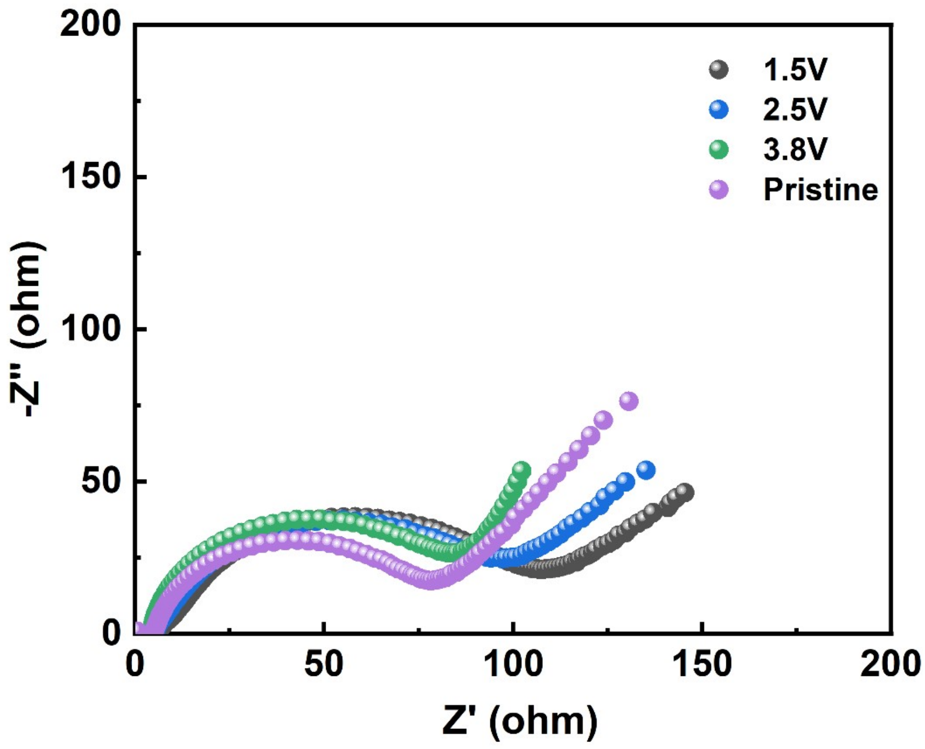

Fig.S7 The EIS plot before cycling of the sodium battery.

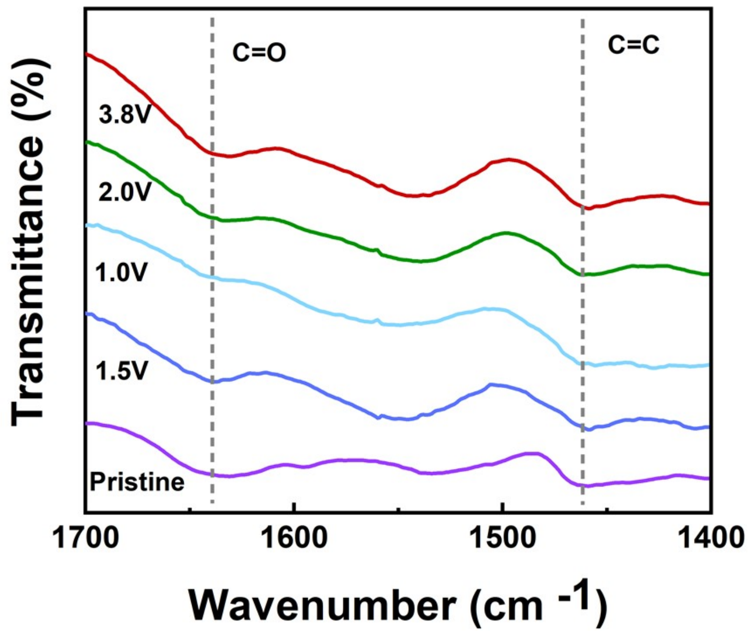

Fig.S8 The ex-situ FTIR spectra of CityU-47.

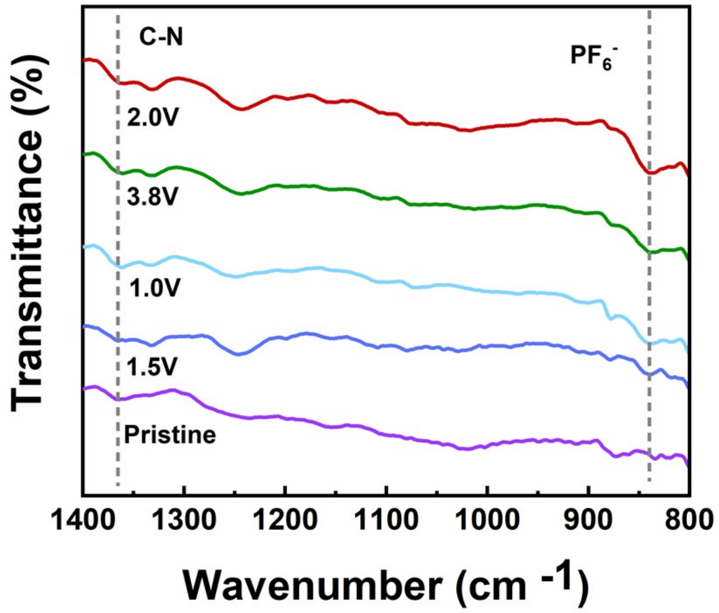

Fig.S9 The ex-situ FTIR spectra of CityU-47.

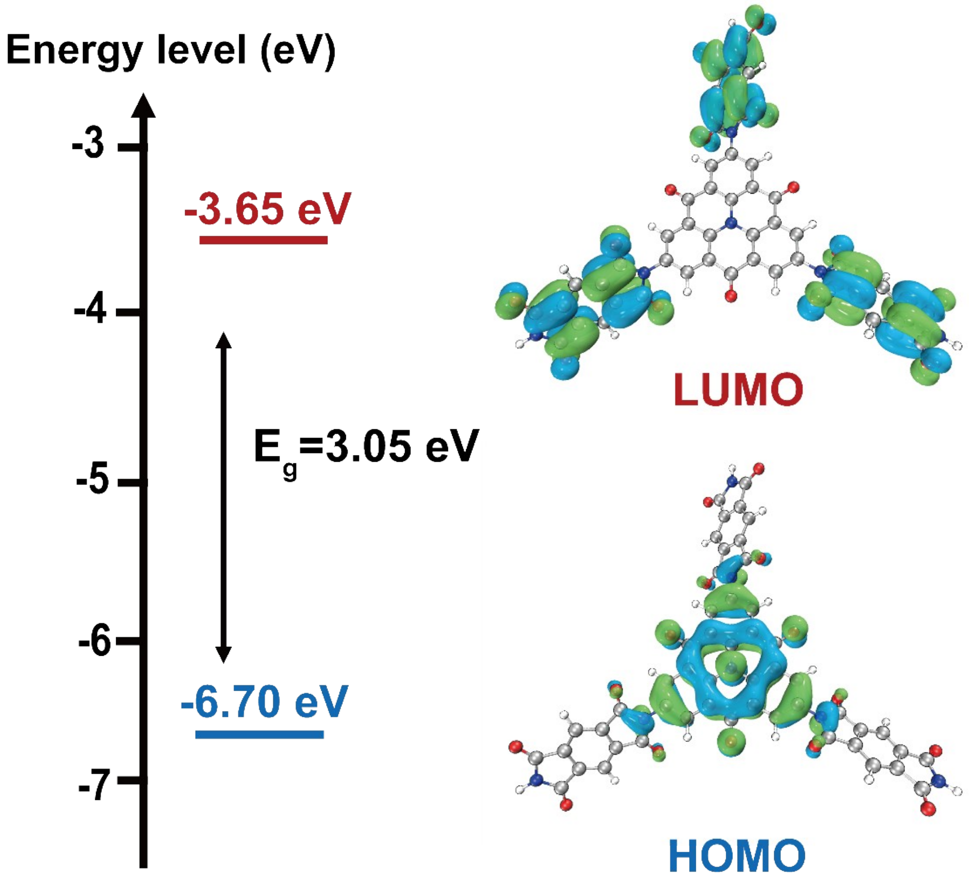

Fig.S10 The distribution of HOMO and LUMO in the building unit of CityU-47.

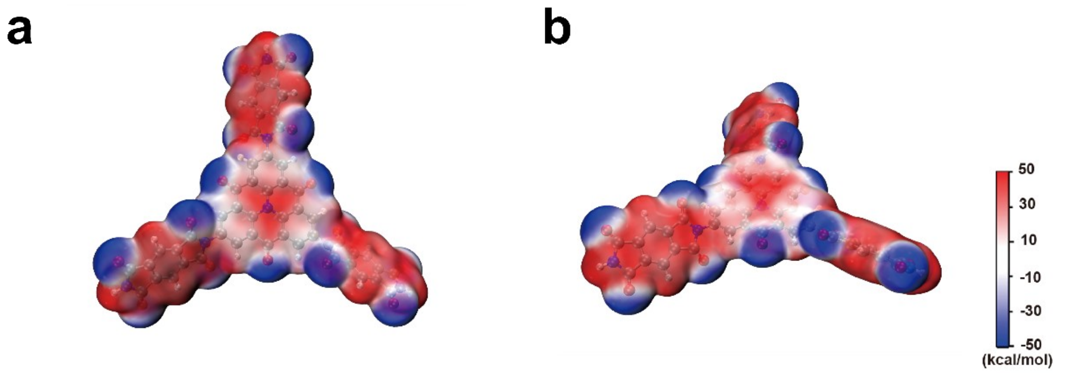

Fig.S11 The molecular electrostatic potential (MESP) distribution in the building unit of CityU-47.

Table S1 Atomic coordinates of the optimized AB-stacking structural model of CityU-2. (space group P1, a = 32.1857Å; b 31.7359Å; c = 3.5839, α = 89.9989°; β = 90.0004°; γ = 120.7255°)

atom label	x/a	y/b	z/c	adp type	occupancy
C1	2.20822	0.62734	0.06934	Uiso	1
C2	2.18465	0.65311	-0.01263	Uiso	1

C3	2.13539	0.62664	-0.10167	Uiso	1
C4	2.10952	0.57523	-0.09988	Uiso	1
C5	2.13332	0.54936	-0.02065	Uiso	1
C6	2.18317	0.57586	0.0597	Uiso	1
N7	2.10679	0.49614	-0.02073	Uiso	1
C8	2.05466	0.46927	-0.06758	Uiso	1
C9	2.13232	0.46979	0.02891	Uiso	1
C10	2.02979	0.49435	-0.13354	Uiso	1
C11	1.97915	0.46839	-0.15321	Uiso	1
C12	1.95238	0.41764	-0.09305	Uiso	1
C13	1.97737	0.39254	-0.04313	Uiso	1
C14	2.02814	0.41779	-0.04245	Uiso	1
C15	2.10727	0.41826	0.00943	Uiso	1
C16	2.13289	0.39335	0.01584	Uiso	1
C17	2.18317	0.41878	0.06654	Uiso	1
C18	2.20733	0.46972	0.11855	Uiso	1
C19	2.18251	0.49538	0.09338	Uiso	1
C20	2.20872	0.54916	0.1302	Uiso	1
C21	2.05412	0.39085	-0.01563	Uiso	1
C22	2.05721	0.548	-0.1744	Uiso	1
O23	2.25146	0.57149	0.21455	Uiso	1
O24	2.03175	0.34611	-0.01399	Uiso	1
O25	2.03662	0.56981	-0.26419	Uiso	1
N26	2.21053	0.70603	-0.00479	Uiso	1
C27	2.25748	0.73718	-0.11191	Uiso	1
C28	2.2703	0.78802	-0.06761	Uiso	1
C29	2.22927	0.78672	0.06651	Uiso	1
C30	2.1927	0.73517	0.10605	Uiso	1
C31	2.31325	0.83218	-0.13983	Uiso	1
C32	2.312	0.87489	-0.06626	Uiso	1
C33	2.27097	0.87358	0.06779	Uiso	1
C34	2.22802	0.82942	0.14002	Uiso	1
C35	2.34857	0.92645	-0.10541	Uiso	1
C36	2.28378	0.92441	0.11245	Uiso	1
O37	2.15227	0.71978	0.23154	Uiso	1
O38	2.28404	0.72389	-0.23912	Uiso	1
O39	2.25718	0.93767	0.23936	Uiso	1
O40	2.38901	0.94185	-0.23097	Uiso	1
N41	2.33075	0.95557	0.00563	Uiso	1
N42	2.88678	0.3921	-0.07391	Uiso	1
C43	2.85918	0.41053	0.05876	Uiso	1
C44	2.80807	0.37364	0.04292	Uiso	1

C45	2.80534	0.33167	-0.09821	Uiso	1
C46	2.85488	0.34433	-0.17229	Uiso	1
C47	2.76688	0.3752	0.14661	Uiso	1
C48	2.7227	0.33149	0.09728	Uiso	1
C49	2.71997	0.28951	-0.04379	Uiso	1
C50	2.76115	0.28795	-0.14749	Uiso	1
C51	2.67317	0.31885	0.17131	Uiso	1
C52	2.66884	0.25263	-0.05952	Uiso	1
O53	2.86647	0.31692	-0.31384	Uiso	1
O54	2.8755	0.45157	0.18714	Uiso	1
O55	2.65251	0.21155	-0.18726	Uiso	1
O56	2.66159	0.34628	0.31269	Uiso	1
C57	2.40865	0.27006	-0.01697	Uiso	1
C58	2.35836	0.24466	-0.06688	Uiso	1
C59	2.33415	0.1937	-0.11825	Uiso	1
C60	2.35893	0.16801	-0.09297	Uiso	1
C61	2.40914	0.19357	-0.02906	Uiso	1
C62	2.43423	0.2451	-0.01046	Uiso	1
N63	2.43462	0.16718	0.0209	Uiso	1
C64	2.40805	0.11396	0.02147	Uiso	1
C65	2.48676	0.19402	0.06726	Uiso	1
C66	2.35819	0.08749	-0.05849	Uiso	1
C67	2.3331	0.036	-0.06787	Uiso	1
C68	2.35665	0.0102	0.01387	Uiso	1
C69	2.40591	0.03666	0.10285	Uiso	1
C70	2.43182	0.08807	0.10091	Uiso	1
C71	2.51159	0.16891	0.13369	Uiso	1
C72	2.56224	0.19483	0.15299	Uiso	1
C73	2.58904	0.24557	0.09212	Uiso	1
C74	2.56408	0.2707	0.04161	Uiso	1
C75	2.51331	0.2455	0.04123	Uiso	1
C76	2.48738	0.27247	0.01387	Uiso	1
C77	2.48412	0.11528	0.1755	Uiso	1
C78	2.33266	0.11421	-0.12901	Uiso	1
O79	2.5098	0.31721	0.01118	Uiso	1
O80	2.50466	0.09347	0.26639	Uiso	1
O81	2.2899	0.0919	-0.21275	Uiso	1
N82	2.64126	0.27108	0.073	Uiso	1
N83	2.33186	0.27028	-0.05805	Uiso	1
C84	2.34833	0.31788	-0.16329	Uiso	1
C85	2.31179	0.33104	-0.09462	Uiso	1
C86	2.27206	0.28951	0.0496	Uiso	1

C87	2.2857	0.25238	0.07296	Uiso	1
C88	2.31207	0.37491	-0.15017	Uiso	1
C89	2.26953	0.37398	-0.04937	Uiso	1
C90	2.22979	0.33244	0.09471	Uiso	1
C91	2.22952	0.28859	0.15036	Uiso	1
C92	2.25588	0.41111	-0.07265	Uiso	1
N93	2.2097	0.39319	0.058	Uiso	1
C94	2.19323	0.34558	0.1631	Uiso	1
O95	2.26049	0.21166	0.20608	Uiso	1
O96	2.38755	0.3448	-0.30644	Uiso	1
O97	2.15397	0.31862	0.30556	Uiso	1
O98	2.28115	0.45189	-0.20489	Uiso	1
H99	2.24606	0.64736	0.14493	Uiso	1
H100	2.11708	0.6461	-0.1746	Uiso	1
H101	1.96058	0.488	-0.21114	Uiso	1
H102	1.95753	0.35344	0.00494	Uiso	1
H103	2.11353	0.35417	-0.02743	Uiso	1
H104	2.24567	0.48966	0.17618	Uiso	1
H105	2.3452	0.83324	-0.24673	Uiso	1
H106	2.19607	0.82837	0.24706	Uiso	1
H107	2.76909	0.4079	0.25927	Uiso	1
H108	2.75895	0.25524	-0.26014	Uiso	1
H109	2.42806	0.30925	0.02571	Uiso	1
H110	2.29579	0.17378	-0.17539	Uiso	1
H111	2.29526	0.01603	-0.14329	Uiso	1
H112	2.4242	0.01717	0.17567	Uiso	1
H113	2.58079	0.17521	0.21127	Uiso	1
H114	2.58396	0.30978	-0.00711	Uiso	1
H115	2.34297	0.40725	-0.26537	Uiso	1
H116	2.19862	0.25625	0.26561	Uiso	1

Reference

(1) F. Kang, L. Yan, Z. Chen, Y. Zhang, Q. Gu, J. Yang, S. Xu, X. Wang, C. S. Lee, Y. Wang and Q. Zhang, *Angew Chem Int Ed* , 2024, e202417779.
